# Five Year Follow Up of Extremely Low Gestational Age Infants after Timely or Delayed Administration of Routine Vaccinations

**DOI:** 10.3390/vaccines9050493

**Published:** 2021-05-12

**Authors:** Ingmar Fortmann, Marie-Theres Dammann, Alexander Humberg, Bastian Siller, Guido Stichtenoth, Geraldine Engels, Janina Marißen, Kirstin Faust, Kathrin Hanke, Sybelle Goedicke-Fritz, Christoph Derouet, Sascha Meyer, Regine Stutz, Elisabeth Kaiser, Egbert Herting, Wolfgang Göpel, Christoph Härtel, Michael Zemlin, on behalf of the German Neonatal Network (GNN) and the PRIMAL Consortium

**Affiliations:** 1 Department of Pediatrics, University of Lübeck, 23538 Lübeck, Germany; dammann@stupa.uni-luebeck.de (M.-T.D.); alexander.humberg@uksh.de (A.H.); bastian.siller@uksh.de (B.S.); guido.stichtenoth@uksh.de (G.S.); kirstin.faust@uksh.de (K.F.); kathrin.hanke@uksh.de (K.H.); Egbert.Herting@uksh.de (E.H.); wolfgang.goepel@uksh.de (W.G.); 2 German Center for Infection Research (DZIF), Partner Site Hamburg-Lübeck-Borstel-Riems, 23538 Lübeck, Germany; 3 Department of Pediatrics, University of Würzburg, 97080 Würzburg, Germany; engels_g@ukw.de (G.E.); marissen_j@ukw.de (J.M.); haertel_C1@ukw.de (C.H.); 4 Department of General Pediatrics and Neonatology, Saarland University, 66421 Homburg, Germany; sybelle.goedicke-fritz@uks.eu (S.G.-F.); christoph.derouet@uks.eu (C.D.); sascha.meyer@uks.eu (S.M.); regine.stutz@uks.eu (R.S.); elisabeth.kaiser@uks.eu (E.K.); michael.zemlin@uks.eu (M.Z.)

**Keywords:** immunization, prematurity, trained immunity, long-term outcome

## Abstract

This study is aimed at detecting the rate of untimely immunization in a large cohort of extremely low gestational age neonates (ELGANs) of the German Neonatal Network (GNN) and at addressing risk factors for delayed vaccination and associated long-term consequences. We performed an observational study of the GNN between 1st January 2010 and 31st December 2019. The immunization status for the hexavalent and pneumococcal immunization was evaluated in *n* = 8401 preterm infants <29 weeks of gestation. Univariate analysis and logistic/linear regression models were used to identify risk factors for vaccination delay and outcomes at a 5-year follow-up. In our cohort *n* = 824 (9.8%) ELGANs did not receive a timely first immunization with the hexavalent and pneumococcal vaccine. Risk factors for delayed vaccination were SGA status (18.1% vs. 13.5%; OR 1.3; 95% CI: 1.1–1.7), impaired growth and surrogates for complicated clinical courses (i.e., need for inotropes, necrotizing enterocolitis). At 5 years of age, timely immunized children had a lower risk of bronchitis (episodes within last year: 27.3% vs. 37.7%; OR 0.60, 95% CI: 0.42–0.86) but spirometry measures were unaffected. In conclusion, a significant proportion of ELGANs are untimely immunized, specifically those with increased vulnerability, even though they might particularly benefit from the immune-promoting effects of a timely vaccination.

## 1. Introduction

Preterm infants are at high risk for infectious diseases early in life, which often require hospital re-admission [1]. Acute respiratory infections, such as pneumonia and bronchitis, are major contributors to morbidity and mortality in this context [2], particularly in extremely low gestational age infants (<29 weeks of gestation; ELGANS) with chronic pulmonary insufficiency after prematurity [3,4]. Early preventive strategies to protect vulnerable infants from infectious diseases include human milk feeding, probiotics and immunizations [5,6,7]. Apart from specific protection against vaccine-preventable diseases, it is discussed whether immunizations in pregnant women (e.g., influenza, pertussis) and postnatal immunizations elicit non-specific immune training effects which contribute to the prevention of infectious diseases in general [7,8,9,10]. The preterm infant’s immune system faces a variety of challenges that predispose them for early and long-term vulnerability. Compared to term infants and adults, the preterm host demonstrates qualitive and quantitative differences in multiple aspects of immunity [11]. Immune development of ELGAN infants lacks important late intrauterine adaptation processes, leading to disturbances in the fragile co-regulation between host defense and immunosuppression (tolerance) [12]. The consequences are an increased risk of adverse short-term outcomes, such as sepsis, and a predisposition to sustained inflammation, both known to imply adverse effects on organ development and long-term vulnerability [13]. Specifically, immune imbalance is characterized by less functional T cells (regulatory T cells), an overexpression of inflammatory cytokines (i.e., IL-1, IL-6, TNF-a) and by its insufficient compensation through immunosuppressive function (i.e., IL-10, S100, regulatory T cells) [12,14]. Mutually influencing mechanisms between the immune system and the infant’s microbiome are also prone to preterm birth-related disturbances (i.e., frequent antibiotic exposure and sepsis) and play a key role in achieving robust and sustainable immunity. When it comes to gut colonization- derived immunity, animal models specifically point at an age-dependent “window of opportunity” to promote optimal immune function [15]. Taken together, studies suggest that in addition to interventions targeting antigen-specific immunity, non-pathogen-specific cell autonomous immunity (CAI), nutritional and innate immune functions can be harnessed to prevent infectious diseases in early and later infancy [16].

In Germany it is recommended for both term and preterm infants, to schedule the first active immunization (hexavalent vaccine: diphtheria, hepatitis type b, tetanus, pertussis, poliovirus, *Haemophilus influenzae type b* and pneumococcal vaccine: 7-, 10- or 13-valent conjugate vaccine) at the age of 2 months after birth [17]. Adherence of neonatal intensive care units (NICU) to these guidelines is required in order to achieve timely immunization of extremely preterm infants. However, historical skepticism on immunogenicity, erroneous assumptions on contraindications, the prolonged accessibility of ELGANs during their primary stay in hospital and other reasons may prompt clinicians to postpone immunizations [18].

Previous data on very low birth weight infants (VLBWI) suggest that timely immunization is associated with a decreased risk of bronchitis in the first year after discharge [6]. The impact of schedule-adherent immunizations for preterm infants has not yet been studied with regard to long-term vulnerability at the age of 5 years. In a large cohort of ELGANs born in the German Neonatal Network (GNN), we addressed the hypothesis that a neonatologist would pay attention to a timely immunization in particular to ELGANs at higher risk for long-term morbidity. However, we found that ELGANs at higher risk for long-term morbidity were less likely to receive a timely vaccination and more frequently suffered bronchitis at the age of 5 years.

## 2. Methods

The German Neonatal Network (GNN) is a population-based observational multicenter cohort study enrolling VLBW infants at 68 neonatal intensive care units (NICU) in Germany. Within the study period, data were collected from infants born between 1st January 2010 and 31st December 2019. Preterm infants of a birth weight <1500 g and a gestational age of 22 + 0 ≤ 28 + 6 weeks, who were actively treated with intensive care were included. To evaluate the effect of timely immunization, only infants who were hospitalized in the timeframe of the recommended first active immunization (primary hospital stay at least 60 days) were included in our study.

After informed written parental consent, a predefined dataset on general neonatal characteristics, antenatal and postnatal treatment and outcome were recorded for each patient on clinical record files at the participating centers. The dataset included the information on whether or not the following immunizations were applied during the primary hospital stay: hexavalent immunization, pneumococcal conjugate vaccine, respiratory syncytial virus (RSV) immunoprophylaxis (palivizumab) and/or rotavirus immunization. After hospital discharge, data sheets were sent to the study center (University of Lübeck). Data quality was evaluated by a physician trained in neonatology via annual on-site monitoring of completed record files. After monitoring, data were coded and evaluated.

Questionnaires concerning the infants’ health status, medical history and treatments were sent to the parents on an annual basis to collect follow-up data. The questionnaires included information on the necessity for re-admissions to hospital, frequency of and reasons for medical visits, medical needs including medication. Further, the occurrence and frequency of bronchitis with or without wheezing and other infectious episodes, e.g., the common cold, were assessed. For the on-site 5-year follow-up, infants were examined by the GNN study team (physician trained in neonatology and 2 study nurses) regarding their motor and cognitive development through standardized tests: Movement Assessment Battery for Children; M-ABC and Wechsler Preschool and Primary Scale of Intelligence—Third Edition; WPPSI I-III. The follow-up included the performance of a hearing test, a visual screening and spirometry. Parents were asked to answer questions concerning the previous medical history and current medical needs as well as to complete a questionnaire concerning detailed information on the children’s social background, illnesses and general development/behavior.

### 2.1. Timely Immunization

“Timely immunized” infants were defined as infants with a primary stay in hospital of at least 60 days who were given both the hexavalent and the pneumococcal immunization. Infants not immunized according to schedule (“not timely immunized”) were hospitalized for at least 60 days but were not immunized with the hexavalent/pneumococcal (heptavalent) immunization during their primary stay in hospital.

### 2.2. Definitions

Gestational age was calculated from the best obstetric estimate based on early prenatal ultrasound and obstetric examination.

Small for gestational age (SGA) was defined as a birth weight less than 10th percentile for gestational age according to sex-specific standards for birth weight by gestational age in Germany [19].

Weight gain velocity was defined as gain in body weight, calculated as gram/day (difference of the parameter at birth and at discharge/number of days in hospital). Growth velocity of body length was defined as gain in body length, calculated as mm/day (difference of the parameter at birth and at discharge/number of days in hospital). Head growth velocity was defined as gain in head circumference, calculated as mm/day (difference of the parameter at birth and at discharge/number of days in hospital). Z-Scores are numerical measurements of the value’s relationship to the mean of the group values measured in terms of standard deviations from the mean (between −3.0 and 3.0). Z-scores were calculated for birth weight according to the 2003 Fenton preterm growth chart.

Bronchopulmonary dysplasia (BPD) was diagnosed when supplemental oxygen or ventilatory support at 36 weeks of postmenstrual age was required. Necrotizing enterocolitis (NEC) was defined as necrotizing intestinal inflammation requiring surgery and focal intestinal perforation (FIP) as FIP requiring surgical treatment classified by the attending surgeon. Periventricular leukomalacia (PVL) was defined as white-matter brain injury, characterized by cystic degeneration of white matter near the lateral ventricles as diagnosed by ultrasound imaging. Intracerebral haemorrhage (ICH) grades I-IV were diagnosed according to the ultrasound criteria of Papile [20]. Retinopathy of prematurity (ROP) was defined as typical retinal changes diagnosed by direct fundoscopy requiring interventions like laser therapy, cryotherapy or intraocular administration of vascular endothelial growth factor inhibitors. Patent ductus arteriosus (PDA) was defined as PDA requiring surgery. Probiotic prophylaxis was defined as exposure to the probiotic formulation consisting of *B. infantis* and *L. acidophilus* corresponding to the formulation that has been most commonly used among the participating study sites in the past.

### 2.3. Statistical Analyses

Data analyses were performed using the SPSS 26.0 data analysis package (IBM, Munich, Germany). Hypotheses in the univariate analysis were evaluated with McNemar’s test, Fisher’s exact test and the Mann–Whitney U test. A *p* value of <0.05 was considered as statistically significant for single tests.

Subsequent to univariate analyses, we included parameters with a *p*-value <0.05 in linear and multivariate logistic regression models and known confounders as independent variables. Accordingly, for the model addressing independent risk factors for vaccination schedule delay we used gestational age, SGA status, ventilation support at discharge, NEC requiring surgery and exposure to inotropes as independent variables (see Table 1 and Table 2). As known confounders for the bronchitis risk or pathological spirometry at 5 years of age, we used gestational age, SGA status, BPD, at least one sibling at home, day care and tobacco smoke as independent variables. Effect size and 95% confidence intervals (CI) were calculated. A *p*-value of <0.05 was considered statistically significant. For primary and subgroup analyses, we used a uniform dataset with available data for all metric parameters. Incomplete datasets were not included.

### 2.4. Ethical Approval

All study parts were approved by the University of Lübeck Ethical Committee and the committees of the participating centers (vote no. 08-022, date of approval: 27th June 2008). Informed consent was obtained from all parents. All methods were carried out in accordance with relevant guidelines and regulations, specifically: the Declaration of Helsinki, the current revision of ICH Topic E6, the Guidelines for Good Clinical Practice, and the Guidelines of the Council for International Organizations of Medical Sciences and the WHO guidelines (“Proposed International Guidelines for Biomedical Research Involving Human Subjects”).

## 3. Results

**Figure 1:**
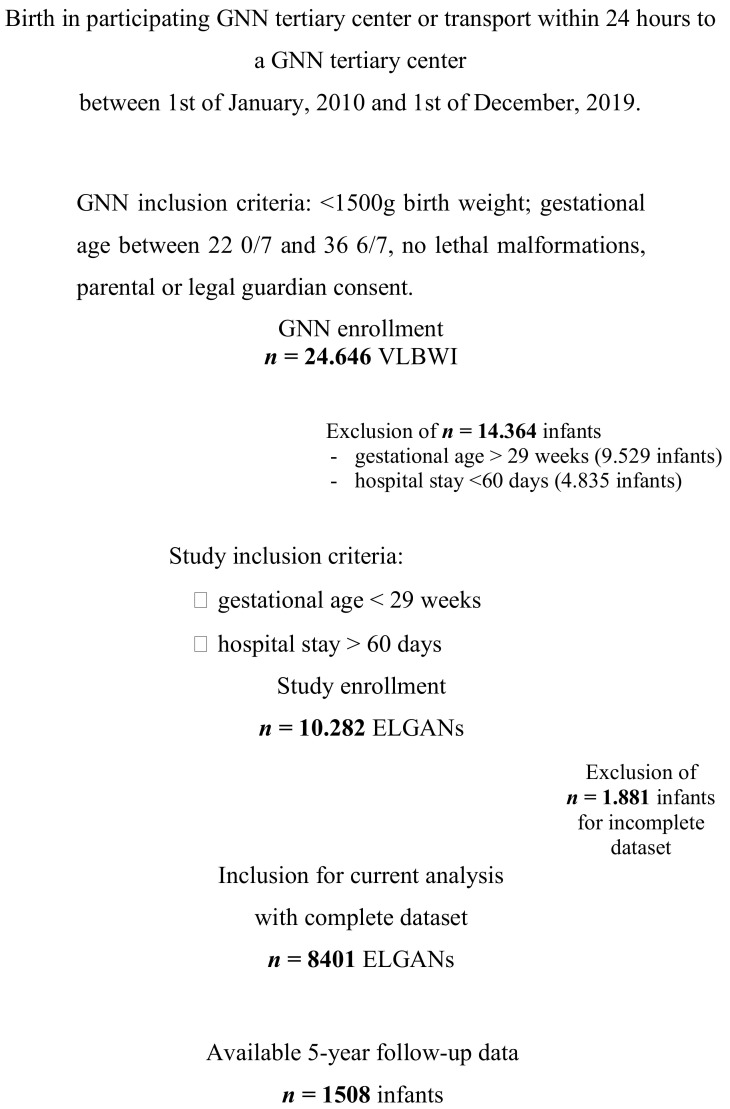
In- and exclusion criteria for the current analysis. GNN, German Neonatal Network; VLBWI, very low birth weight infant; ELGAN, extremely low gestational age neonate.

A total of *n* = 8401 ELGANs met the inclusion criteria within the study period (Figure 1). The study cohort had a mean gestational age at birth of 26.3 weeks (median 26.4 w; SD 1.6 w) and a mean birth weight of 821 g (median 810 g; SD 227 g, Table 1); 53.1% of the infants were male, 33.2% multiples and 13.7% were small for gestational age (SGA). During their primary stay, infants were hospitalized for a mean of 97.9 days (median 90.0 d). N = 7577 infants (90.2%) received a timely first active immunization with a hexavalent immunization and pneumococcal conjugate vaccine whereas *n* = 824 infants (9.8%) had no schedule-adherent immunization.

### 3.1. SGA Infants Are Less Often Immunized According to Schedule

As outlined in Table 1, timely immunized infants showed a lower gestational age (26.3 w vs. 26.5 w; *p* < 0.001), had a higher gestational age at discharge (40.3 w vs. 40.0 w; *p* < 0.001), were longer hospitalized during their primary stay (mean, 98.2 d vs. 94.7 d; *p* < 0.001) and were less often born to a mother of Caucasian ethnicity (70.3 vs. 73.9%, *p* = 0.006) than infants in the group of no timely immunization. Notably, infants who did not receive a timely first immunization were significantly more often SGA (18.1% vs. 13.5%; *p* < 0.001) and had lower growth velocities of body weight (g/d; 19.9 vs. 21.6; *p* = 0.005), head circumference (mm/d; 0.99 vs. 1.02; *p* < 0.001) and body length (mm/d; 1.3 vs. 1.4; *p* < 0.001). Likewise, these infants presented with a lower body weight at discharge including Fenton Z-scores (−1.76 vs. −1.43; *p* < 0.001) and lower weight gain during the hospital stay (Fenton Z-scores; −1.23 vs. −0.99; *p* < 0.001).

### 3.2. Severe Clinical Courses Coincide with Delay in Immunizations

Univariate analyses revealed that non-timely immunized ELGANs had more often been exposed to invasive treatments such as inotropes (10.4 vs. 7.5%; *p* = 0.002), surgery for various reasons (40.1 vs. 36.1%; *p* = 0.025) or inhaled nitric oxide (9.4 vs. 7.4; *p* = 0.041) and had a longer duration of mechanical ventilation (median 5.0 vs. 4.0 days; *p* < 0.001; Table 2) than infants that were immunized timely. We also noted that a delay in immunizations coincided with a higher rate of pneumothoraces, ventilation support at discharge (15.0 vs. 2.9%; *p* < 0.001) and supplemental oxygen at discharge (18.1 vs. 8.6%; *p* < 0.001). The disease burden of the “not-timely immunized” group was characterized by a higher incidence and higher adjusted risk (adjusted for gestational age and SGA-status) for periventricular leukomalacia (6.8% vs. 4.2%; *p* = 0.001; adjusted OR: 1.7, 95% CI: 1.3–2.4), intraventricular hemorrhage (30.0% vs. 26.9%; *p* = 0.049; adjusted OR: 1.3; 95% CI: 1.1–1.5) and NEC requiring surgery (6.6% vs.3.6%, *p* < 0.001; adjusted OR 2.0; 95% CI: 1.5–2.8) but not for ROP, FIP or PDA requiring surgery.

### 3.3. Risk Constellations for Delay in Immunizations

Next, we evaluated parameters that might impact on the delay of immunization timing in a multivariate logistic regression model. We found that SGA status (OR 1.3; 95% CI: 1.1–1.7), ventilation support at discharge (OR 3.1; 95% CI: 1.9–4.2), NEC requiring surgery (OR 1.9; 95% CI: 1.4–2.8) and exposure to inotropes (OR 1.5; 95% CI: 1.1–2.1) were associated with the neonatologist’s decision not to immunize according to the guideline. In addition, we adjusted the model for other surgery indications such as PDA, ROP, FIP and hydrocephalus requiring surgery (VP-shunt OR 1.7; 95% CI 1.2–2.6); SGA status and surrogate measures for severe clinical course (inotropes, ventilation support at discharge) proved to be independent risk factors for immunization delay.

In our study cohort of 8401 ELGAN infants we noticed that, interestingly, those infants that were not vaccinated according to schedule more often had mothers of German descent when compared to infants without timely immunization (73.9% vs. 70.3%, *p* = 0.006), a finding that could be confirmed in the subgroup of infants with a 5-year follow-up (86.7% vs. 78.7%, *p* = 0.02). Timely immunized infants were more often from the rest of Europe or Africa in the neonatal cohort and more frequently from Asia and Africa in the follow-up cohort. Our study is limited by lacking information on the reasons for untimely vaccination, which, in this context, could be cultural differences with regard to vaccination hesitancy or barriers of language.

### 3.4. Non-Timely Immunized Infants Have an Increased Bronchitis Risk at the Age of 5 Years

**Figure 2:**
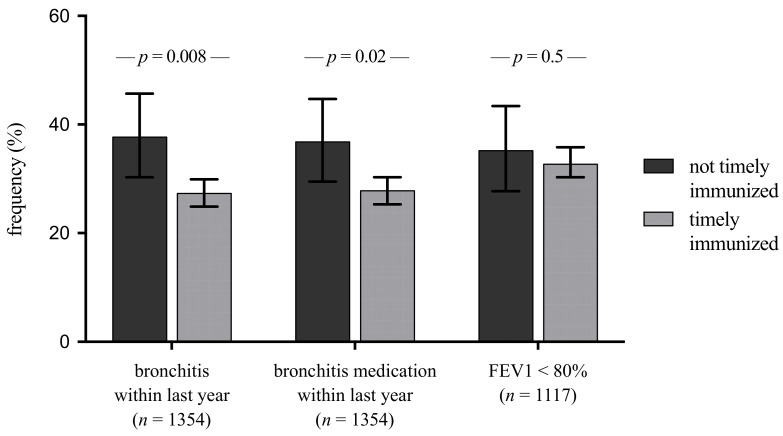
Outcome at 5 years of age: parent-reported bronchitis (medication) within last year and pathological FEV1. FEV1, forced expiratory volume in 1 s; *p*-values were derived from Pearson’s Chi-square test.

**Figure 3:**
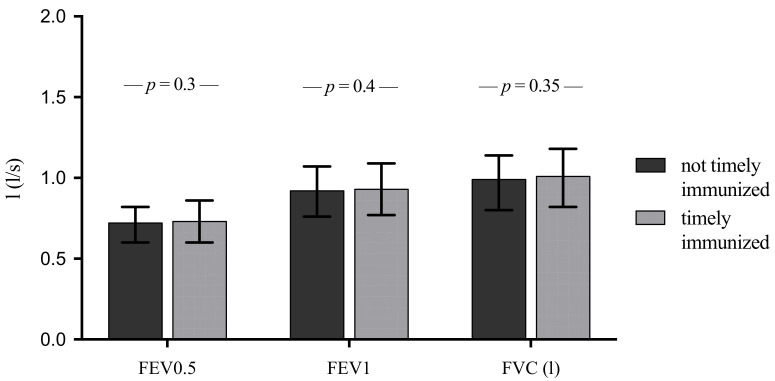
Spirometry at 5-year follow-up. FEV0.5/1, forced expiratory volume in 0.5/1 s/s; FVC, forced vital capacity; *p*-values were derived from Pearson’s Chi-square test; *n* = 1117 ELGANs.

Non-timely immunized infants had more parent-reported episodes of bronchitis at the age of 5 years than timely immunized infants (2.11 vs. 1.17, *p* = 0.01; Table 3) in univariate analyses. Children that had been timely immunized had a decreased risk of bronchitis at 5 years of age (logistic regression, OR = 0.60; CI: 0.42–0.86) and the frequency of overall bronchitis episodes (linear regression, effect size B = −0.09; 95% CI: −1.36–−0.1) independent of BPD, gestational age, SGA status, sibling- and smoke- exposure at home and daycare (Figure 2). In order to address heterogeneity in the quality of care between the centers or regional risk factors for bronchitis (i.e., air pollution), we added facility-fixed effects to our analyses. We still found a negative, statistically significant correlation between timely immunization and the occurrence of bronchitis within the 5th year (OR 0.56; 95% CI 0.3–0.9; *p* = 0.01) and the use of bronchitis medication within the last year at the age of 5 years (OR 0.57; 95% CI 0.35–0.9; *p* = 0.02), whereas the negative correlation between timely immunization and number of bronchitis episodes didn´t reach statistical significance (regression coefficient B −0.606; 95% CI −1.5–0.3; *p* = 0.19). Likewise, we added year-fixed effects to our analyses in order to address different vaccines used throughout the year (i.e., PCV-7, PCV-10 and PCV-13). We found a negative, statistically significant correlation between timely immunization and the occurrence of bronchitis within the 5th year (OR 0.6; 95% CI 0.4–0.9; *p* = 0.025) and the use of bronchitis medication within the last year at the age of 5 years (OR 0.63; 95% CI 0.4–0.9; *p* = 0.024), whereas the negative correlation between timely immunization and number of bronchitis episodes didn´t reach statistical significance (regression coefficient B −0.338; 95% CI −1.13–0.45; *p* = 0.4). In terms of spirometry parameters at the 5-year follow-up, such as forced expiratory volume in 0.5 s (FEV0.5), forced expiratory volume in 1 second (FEV1) or forced vital capacity (FVC), there was no notable influence of timely vaccination (hexavalent, pneumococcal and heptavalent; Figure 3.

General clinical characteristics of the cohort of infants with a 5-year follow-up are displayed in Supplementary Tables S1 and S2. We found minor differences in gestational age (26.8 weeks vs. 26.3 weeks) and birth weight (863 g vs. 816 g) and no differences in general clinical characteristics and main neonatal outcomes, such as IVH, NEC, PDA, FIP, BPD, etc. These data support the view that the increased risk of timely vaccinated infants for bronchitis is not biased by general clinical characteristics and main neonatal outcomes, in particular by BPD or NEC. Supplementary Table S2 demonstrates that there were no significant differences between the groups with regard to body weight, BMI and head circumference at the age of 5 years. There was a trend towards an improved growth in length in the timely immunized group (*p* = 0.03). Further, we tested our results of the 5-year follow-up for gender distribution across the subgroups and found no significant differences (bronchitis within last year, untimely vaccinated: males vs. females 42.2% vs. 32.4%, *p* = 0.22; timely vaccinated males vs. females 28.2% vs. 26.4%, *p* = 0.49). In both the timely and non-timely vaccinated infants, male subjects had a trend towards a higher rate of bronchitis within the last year. This trend was more pronounced among the non-timely vaccinated infants but did not show significance (*p* = 0.22).

## 4. Discussion

In a large population-based cohort of ELGANs we noted a significant number of infants who had not been immunized according to the recommended schedule. Associated risk factors for lacking immunization at discharge from a primary hospital stay of 60 or more days were SGA status and surrogates for complicated courses (need for inotropes, surgery for NEC, ventilation support at discharge.). At 5 years of age, delayed immunization was associated with an increased risk of bronchitis but not with reduced spirometry measures.

International guidelines recommend administering immunization in preterm infants according to the same schedule as term infants [17,21,22]. A delay is frequently reported in this population [23,24,25], which can be confirmed by our observational data from German NICUs for approximately 10% of infants. We observed a mean schedule delay of at least 35 days in non-timely immunized infants. In contrast to our hypothesis, preterm infants at increased risk of infectious diseases were not immunized as consistently as term infants. We speculate that the reasons include lack of knowledge and uncertainty of parents and healthcare professionals about the tolerability and efficacy of vaccinations in infants with higher prematurity-related risks for long-term morbidity [26,27]. The safety and effectiveness of immunization in preterm infants has been confirmed several times [21,28,29]. Historical skepticism towards schedule-based immunization in preterm infants might be related to the clinical aspects of increased vulnerability in ELGAN infants including SGA status, reduced muscle mass and surrogate measures of complicated courses, i.e., invasive treatments and requirement of prolonged medical support such as oxygen need at discharge. One might speculate that clinicians are reluctant to administer another “inflammatory stimulus” to a susceptible individual [12]. However, these infants represent the subgroup with the highest risk of preventable infectious diseases and therefore might specifically benefit from timely immunization. In addition, our observational data do not support arguments to withhold timely immunizations in ELGANs with ROP (due to concerns of aggravation of the disease by the vaccination responses). Surgery for various reasons, such as NEC, stoma repositioning or PDA may be another reason for clinicians to postpone immunization of ELGAN infants. Three days prior to surgery, immunization may be avoided in order to accomplish optimal medical conditions without vaccine-related cardiorespiratory instability. After surgery, infants have to be carefully monitored for fever as a sign of surgery-related infectious complications, therefore a critical timeframe to withhold immunizations following surgery needs to be individually determined.

Immunizations on the NICU implicate the opportunity for physicians and care givers to actively influence the immunological maturation of preterm infants. At 5 years of age, children that had obtained a delayed first immunization had a higher risk of bronchitis, which may serve as a surrogate measure for increased pulmonary susceptibility. We herein hypothesize that the increased vulnerability shown by our data is not related to BPD or spirometry measures, but rather based on immune-functional inferiority compared to timely immunized infants. It is known that the vulnerability of the preterm infant to infections extends beyond the perinatal period into young infancy and adolescence [30]. Specifically, reduced abundances of CD4+ cells, increased levels of proinflammatory cytokines and T helper cell cytokines, such as IL-4 and IL-13 can still be detected in late childhood [31]. Various immunological mechanisms may contribute to increasing the risk for bronchitis and/or wheezing. First, long-term adverse outcomes of preterm infants might include a reduced immune function leading to an increased risk of viral infection and bronchitis, leading to hospital re-admissions and increased risk of lung alteration. Detrimental trained immunity-effects in non-timely vaccinated preterm infants may possibly result in an impaired concept of long-term adaptation of innate immune cells (32). The underlying mechanisms of trained immunity are very diverse and include: non-antigen specific priming of natural killer cells, programming of myeloid cells, chromatin modifications associated with gene silencing of inflammatory pathways, alterations of chromatin marks in monocytes, proinflammatory cytokine responses (especially IFN-γ production), that are crucial for immunological memory responses [32]. The second immune-mediated phenomenon results in an “asthma phenotype”, that is possibly but not necessarily triggered by infectious stimuli. Preterm birth along with increased rates of cesarean section, sepsis and gastrointestinal dysbiosis promotes the development of this clinical phenotype. Our observations support the hypothesis that immunization according to schedule fosters immunological maturation to protect from vaccine-preventable disease [7] but also from the overall infectious disease burden, including upper and lower airway viral infections leading to hospital re-admissions. This is in line with a non-specific reduction of mortality caused by immunization, that has been previously described in animal models as well as for preterm humans [33]. Future studies need to focus on a better comprehension of prematurity-related long-term effects on immunity, that promote therapeutic and preventive strategies—including immunizations—to reduce the risk of immune-mediated diseases. Preventive strategies that protect infants at risk from infections throughout young infancy, may have implications for long-term pulmonary vulnerability, such as asthma and even pulmonary fibrosis as recent studies have suggested [34]. Long-term follow-up of preterm infants may disentangle the context between early prevention of specific respiratory infection, unspecific trained immunity effects of timely immunizations and long-term lung morbidity.

### Strengths and Limitations

The main strengths of our study design are the large sample size, on-site monitoring of the clinical data by neonatology-trained staff as well as standardized follow-up examinations performed by the same physicians and observer team. Our study provides a benchmarking analysis for educational toolkits for health care professionals and parental counselling.

There are various limitations to our data. We do not have information on the immunization schedule after discharge. Whether and when non-immunized infants received their first immunization after discharge and if timely immunized infants received further immunization according to schedule is unknown. The risk of bronchitis is based on parental questionnaires and could not be adjusted for an infectious season. Further, we have no information on the reasons why caregivers decided to delay or not to vaccinate in the recommended timeframe. Finally, it is unclear whether the increased risk of infants that were not timely immunized was caused by their pre-existing risk factors or by the delay of their vaccination, or both. Future studies need to promote prospective approaches with detailed documentation on preterm infants’ vaccinations, including vaccine types and dates as well as perform extensive follow-up investigations including detailed information on infection susceptibility and pathogen-related pulmonary outcomes.

## 5. Conclusions

A significant proportion—almost 10% of ELGAN infants—are not timely immunized according to the nationally and internationally recommended schedules. Our cohort is already characterized by a high prematurity-related vulnerability—and therefore benefits greatly from infection-preventive measures, such as immunizations. However, among these infants, the subgroup with the highest susceptibility due to growth impairment and complicated clinical courses, are particularly affected by schedule delay. Education of parents and healthcare providers about safety, tolerability, efficacy, immunogenicity and long-term benefits is necessary in order to improve the rate of timely vaccinations in the most vulnerable preterm neonates since they might benefit from direct and indirect immune-promoting effects of timely vaccination more than healthier ELGANs.

## Figures and Tables

**Table 1:** Clinical characteristics stratified to timely pneumococcal and hexavalent immunization.

	No Timely Immunization (*n* = 824; 9.8%)	Timely Immunization (*n* = 7577; 90.2%)	*p*	Total (*n* = 8401)
Gestational age (weeks)	26.5 1.7 (26.7)	26.3 1.5 (26.4)	<0.001 ^#^	26.3 1.6 (26.4)
Birth weight (g)	834 253 (840)	820 224 (810)	0.11	821 227 (810)
Birth weight (Z-score, Fenton)	−0.31 0.97 (−0.2)	−0.23 0.89 (−0.13)	0.06	−0.27 0.9 (−0.2)
Multiples (%)	31.8	33.3	0.37	33.2
Male gender (%)	55.5	52.9	0.2	53.1
SGA (%)	18.1	13.5	<0.001	13.7
Gestational age at discharge (weeks)	40.0 7.9 (38.7)	40.3 4.1 (39.3)	<0.001 ^#^	40.3 4.6 (39.2)
Age at discharge (d) (length of primary stay in hospital)	94.7 31.4 (81.0)	98.2 33.4 (90.0)	<0.001 ^#^	97.9 36.7 (90.0)
Growth velocity (g/d)	19.9 5.5 (20.3)	21.6 4.3 (21.6)	0.005 ^#^	21.4 4.3 (21.7)
Head growth velocity (mm/d)	0.99 0.23 (0.96)	1.02 0.19 (1.03)	<0.001 ^#^	1.02 0.2 (1.02)
Growth velocity of body length (mm/d)	1.3 0.4 (1.4)	1.4 0.3 (1.4)	<0.001 ^#^	1.4 0.3 (1.4)
Body weight at discharge (Z-score, Fenton)	−1.8 1.07 (−1.64)	−1.4 0.88 (−1.39)	<0.001 ^#^	−1.5 0.9 (−1.42)
Weight gain (Z-score, Fenton)	−1.2 1.05 (−1.16)	−0.99 0.91 (−0.98)	<0.001 ^#^	−1.01 0.9 (−1.42)
Probiotic prophylaxis (%)	66.5	79.4	<0.001	78.2
Human milk (%)	80.5	83.3	0.07	83.0
Formula (%)	56.2	70.1	<0.001	68.9
Maternal descent: Caucasian (Germany, %)	73.9	70.3	0.006	70.7
Other Europ. countries, incl. Russia (%)	9.3	12.5	0.008	12.2
Africa (%)	2.8	4.3	0.03	4.2
Middle East/Turkey (%)	9.4	9.5	0.1	9.5
Asia (%)	2.9	2.5	0.2	2.5
Other Europ. countries, incl. Russia (%)	9.3	12.5	0.008	12.2

SGA, small for gestational age (<10th Voigt percentile); *p*-values were derived from chi-square test if not otherwise indicated (^#^, Mann–Whitney U test). Continuous variables and Z-scores are shown as mean/SD (median).

**Table 2:** Treatment and outcome parameters stratified to timely pneumococcal and hexavalent immunization.

	No Timely Immunization (*n* = 824; 9.8%)	Timely Immunization (*n* = 7577; 90.2%)	*p*	Total (*n* = 8401)
Pneumothorax (%)	10.0	5.2	<0.001	6.1
Inhaled NO (%)	9.4	7.4	0.041	7.6
Duration of ventilation (d)	15.3 24.4 (4.0)	11.6 17.5 (5.0)	<0.001 ^#^	12.3 19.3 (5.0)
Inotropes (%)	10.4	7.5	0.002	7.7
Oxygen need at discharge (%)	18.1	8.6	<0.001	9.5
Ventilation support at discharge (%)	15.0	2.9	<0.001	4.1
IVH (%)	30.0	26.9	0.049	27.2
PVL (%)	6.8	4.2	0.001	4.4
Any surgery (%)	40.1	36.1	0.025	36.5
NEC (%)	6.6	3.6	<0.001	3.9
FIP (%)	5.1	4.5	0.47	4.6
ROP (%)	4.8	4.8	0.9	4.8
PDA (%)	6.6	7.1	0.58	7.0

NO, nitric oxide; IVH, intraventricular hemorrhage; PVL, periventricular leukomalacia; NEC, necrotizing enterocolitis requiring surgery; FIP, focal intestinal perforation; ROP, retinopathy of prematurity with intervention; PDA, patent ductus arteriosus with intervention, any surgery includes interventions for NEC, FIP, PDA and ROP; *p*-values were derived from chi-square test if not otherwise indicated (^#^, Mann–Whitney U test). Continuous variables are shown as median/mean/SD (median).

**Table 3:** Timely immunization is protective for bronchitis risk at 5 years.

Outcome	No Timely Immunization	Timely Immunization	Adjusted OR * (95% CI) for Outcome
Bronchitis episodes (n/month 48–60)	2.11 2.62 (1.0)	1.17 2.10 (1.0)	* B = −0.09 (−1.4–−0.1); *p* = 0.04
Bronchitis within last year (%)	37.7	27.3	^#^ OR 0.60 (0.4–0.8); *p* = 0.006
bronchitis medication within last year (%)	36.8	27.8	^#^ OR 0.63 (0.4–0.9); *p* = 0.01
FEV1 <80% (%)	35.4	32.4	^#^ OR 0.79 (0.5–1.2); *p* = 0.2

Linear (*) und logistic (^#^) regression analyses are adjusted for known confounders: gestational age, SGA status, BPD, sibling(s) at home, day care, smoke exposure; *n* = 1354 infants had a complete dataset with parental interviews and *n* = 1117 infants had spirometry testing at 5-year follow-up; continuous variables are shown as median/mean/SD (median).

## Data Availability

The data that support the findings of this study are available from the corresponding author, [I.F.], upon reasonable request.

## Supplementary Materials

The following are available online at https://www.mdpi.com/article/10.3390/vaccines9050493/s1, Table S1: Clinical and outcome characteristics of infants with 5-year follow-up. Table S2: Growth parameters of infants with 5-year follow-up.
