# Supplementary material for: Five Year Follow Up of Extremely Low Gestational Age Infants after Timely or Delayed Administration of Routine Vaccinations
Source: Vaccines (Basel). 2021 May 12;9(5):493. doi: 10.3390/vaccines9050493 (PMC8150373; doi:10.3390/vaccines9050493)
Supplement: Supplementary file 1 [file vaccines-09-00493-s001.zip › vaccines-1126131-supplementary.pdf]

vaccines

MDPI

Supplementary Table S1. Clinical and outcome characteristics of infants with 5-year follow-up.

	No timely immunization (n=160; 11.8%)	Timely immunization (n=1194; 8.2%)	p	Total (n= 1354)
**Gestational age** (weeks)	26.8<br>1.5 (27.0)	26.3<br>1.5 (26.4)	<0.001#	26.3<br>1.5 (26.4)
**Birth weight** (g)	863<br>239 (855)	816<br>222 (803)	0.008#	821<br>224 (810)
**Multiples** (%)	34.1	37.2	0.4	36.9
**Male gender** (%)	44.3	47.8	0.4	47.4
**SGA** (%)	16.8	14.5	0.5	14.8
**Maternal descent:**				
**Caucasian (Germany**, %)	86.7	78.7	0.02	79.7
**Other Europ. countries, incl. Russia** (%)	5.1	9.3	0.1	8.8
**Africa (%)**	1.2	4.6	0.04	4.3
**Middle East/Turkey (%)**	5.1	5.3	0.1	5.3
**Asia**	0.6	1.4	0.08	1.2
**IVH (%)**	26.3	24.9	0.7	25.1
**PVL (%)**	4.8	3.6	0.4	3.8
**NEC (%)**	3.0	3.4	0.7	3.3
**FIP (%)**	1.8	4.8	0.07	4.4
**BPD (%)**	27.5	33.7	0.1	33.0
**PDA (%)**	6.0	7.8	0.4	7.5
**CP (5 years, %)**	7.9	6.9	0.6	7.1

Legend: SGA, small-for-gestational-age; IVH, Intraventricular hemorrhage; PVL, Periventricular leukomalacia; NEC, Necrotizing Enterocolitis requiring surgery; FIP, Focal intestinal perforation; BPD, Bronchopulmonary Dysplasia; PDA, Patent ductus arteriosus with intervention; Cerebral Palsy; p-values were derived from chi-square test if not otherwise indicated (#, Mann-Whitney-U test). Continuous variables are shown as median/mean/SD (median).

Supplementary Table S2. Growth parameters of infants with 5-year follow-up.

	No timely immunization (n=160; 11.8%)	Timely immunization (n=1194; 8.2%)	p	Total (n= 1354)
**Birth weight** (g)	863<br>239 (855)	816<br>222 (803)	0.008	821<br>224 (810)
**Body length**<br>(at birth, cm)	34.3<br>3.4 (34.0)	33.5<br>3.2 (33.5)	0.005	33.6<br>3.2 (24.0)
**Head circumference**<br>(at birth, cm)	24.2<br>2.1 (24.5)	23.6<br>2.1 (23.7)	<0.001	23.7<br>2.1 (24.0)
**Body weight** (5 years, kg)	18.4<br>4.3 (17.6)	18.6<br>3.5 (18.0)	0.08	18.6<br>3.6 (18.0)
**Body length** (5 years, cm)	111.7<br>6.8 (111.1)	112.7<br>6.2 (112.8)	0.03	112.6<br>6.2 (112.6)
**BMI** (5 years, kg/m^2**)**	14.6<br>1.9 (14.3)	14.5<br>1.7 (14.3)	0.9	14.5<br>1.7 (14.3)
**Head circumference**<br>(5 years, cm)	49.8<br>2.2 (50.0)	50.0<br>2.2 (50.0)	0.88	49.9<br>2.2 (50.0)

Legend: BMI Body-Mass-Index; p-values were derived from Mann-Whitney-U test. Continuous variables are shown as median/mean/SD (median).
